# Clinical re-evaluation of removing blood stasis therapy in treating acute intracerebral hemorrhage safety and efficacy: a protocol for a randomized, controlled, multicenter study (CRRICH Trial)

**DOI:** 10.1186/s40064-016-3136-y

**Published:** 2016-09-01

**Authors:** Liling Zeng, Jianwen Guo, Jing Wang, Qixin Zhang, Haijun Li, Rongming Lin

**Affiliations:** 1 No. 1 Neurology Department, The 2nd Teaching Hospital of Guangzhou University of Chinese Medicine, Guangdong Provincal Hospital of Chinese Medicine, Guangdong Province Key Laboratory of Emergency medicine of TCM., 111 Da’de Road, Yuexiu District, Guangzhou, Guangdong China; 2 Neurology Department, Shenzhen Longhua New District Center Hospita, Shenzhen, China

**Keywords:** Intracerebral hemorrhage, Clinical trial, Traditional Chinese medicine, Promoting blood circulation, Removing blood stasis

## Abstract

**Background:**

Hypertensive intracerebral hemorrhage (HICH) is one of the most devastating forms of stroke. Currently, no specific therapies for HICH except general medical care. However, in China, medicine of promoting blood circulation (PBC) and removing blood stasis (RBS) are widely and efficiently used to treat HICH and become a potentially effective treatment for the secondary effects of HICH to alleviate brain injury, accelerate neuronal recovery, and improve the prognosis. In order to evaluate the safety and effect of PBC and RBS herbal drugs, we design a prospective, randomized, open, double-blind controlled clinical trial on the hematoma enlargement in HICH patients treating with PBC and RBS herbal medicine within 6 h time window from the symptom onset.

**Methods/design:**

A multicenter, three-group, prospective, randomized, double-blinded and placebo-controlled clinical trial. Patients aged 18 or older with HICH confirmed by CT scan within 6 h from the onset are included. 360 patients will be randomized to 3 groups (PBC & RBS & Placebo) within 6 h of ictus. Stratified block randomization is undertaken using a sequentially numbered and opaque envelope. All subjects must take medicine within 6 h of ictus and have another CT scan at about 24 h to confirm hematoma expansion. A postal questionnaire to the patients to evaluate their recorvery at 3 months. Primary outcome is the percent change in the volume of hematoma at 24 h. Secondary outcomes include: mortality, disability, serious adverse events, etc.

**Conclusions:**

The CRRICH Trial is expected to confirm the safety and effect of acute intracerebral hemorrhage treated within 6 h of ictus with “RBS” therapy and to determine whether the traditional therapy can cause hematoma growth after intracerebral hemorrhage.

**Discussions:**

This is the first  prospective, multicenter, randomized, placebo-controlled clinical trial to investigate herbal medicine whether can induce the incidence of hematoma enlargement of AICH patient within the 6 h time window from onset. We need the data to keep the herbal clinical usage safety.

*Trial registration* clinicaltrials.gov: NCT01918722

## Background

HICH is one of the most devastating forms of stroke which is a main cause of death and disability around the world (Roach et al. 2005), playing a significant part in morbidity and mortality worldwide (Qureshi et al. 2009). The proportion of patients with ICH who died within 30 days has risen to nearly 40 %, and most of the survivors cannot make a full recovery (Qureshi et al. 2009; van Asch et al. 2010). Approximately two-third of ICH patients suffer hematoma enlargement within 24 h of ictus (Lim et al. 2008). Moreover, hematoma growth is an independent prognostic determinant (Davis et al. 2006). Several reasons may be related to the hematoma enlargement in the early stage of ICH, including high blood pressure, “spot” sign of CT scan, sex, age, time window, anticoagulation drugs (Lim et al. 2008). But so far there have not been specific therapies or treatments preventing hematoma expansion and improve the outcome after ICH. Furthermore, it has been confirmed that neither mini-traumatic operation nor the present accessible medicine is proved to be the ideal treatment based on the available evidence-based medical researches (Morgenstern et al. 2010; Mendelow et al. 2013; Mayer et al. 2008).

Faced with the limitations of the current available therapies, herbal medicine of promoting blood circulation (PBC) and removing blood stasis (RBS) are widely and efficiently used in Chinese hospitals to treat HICH based on the TCM theory of ‘the blood flow outside the vessels is the blood stasis’. However, whether these herbal medicine can cause hematoma enlargement leading to serious adverse events is undefined until now (Li et al. 2015).

In order to evaluate the safety of PBC and RBS herbal drugs, we design a prospective, randomized, open, double-blind controlled clinical trial on the hematoma enlargement in HICH patients treating with PBC and RBS herbal medicine within 6 h time window from the symptom onset.

## Study design and methods

### Objective

 The aim of this study is to evaluate the safety and effect of acute intracerebral hemorrhage (AICH) treated with “RBS” therapy and to determine whether the traditional therapy can cause hematoma growth after intracerebral hemorrhage.

### Centre eligibility

There are 13 centers included from all over China. Only hospitals that have been experienced in clinical trial and previously abided by trial guidelines well are qualified to be research centers.

### Design

**Fig. 1:**
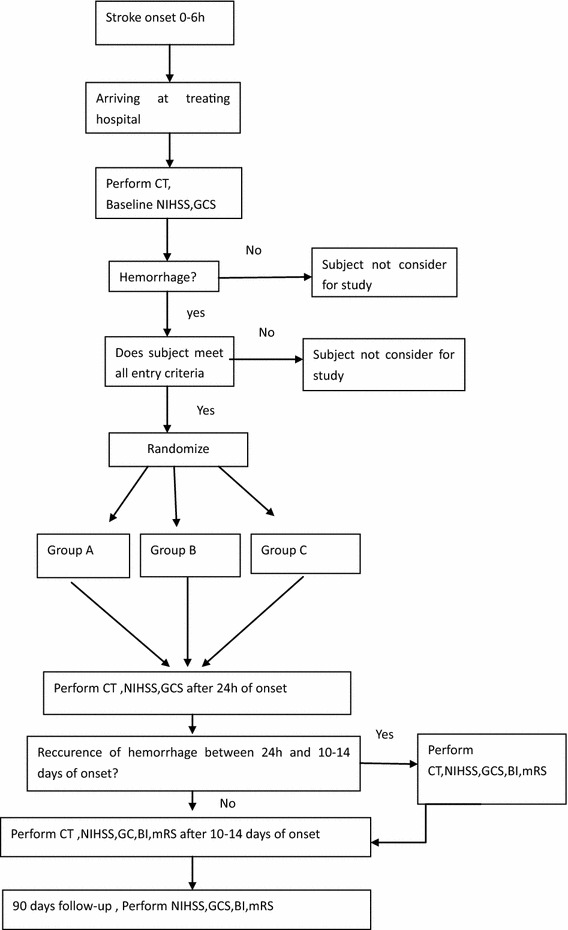
Study design diagram. *CT* computed tomography, *CTA* computed tomography angiography, *NIHSS* National Institutes of Health Stroke Scale, *mRS* modified Rankin Scale, *BI* Barthel Index, *GCS* Glasgow Coma Scale

A multicenter, three-group, prospective, randomized, double-blinded and placebo-controlled clinical trial. The study patient flow diagram is displayed in Fig. 1.

### Patient population

Entry criteria will be structured to enroll patients aged 18 or older with ICH confirmed by CT scan within 6 h after the onset. The exclusion criteria are surgical evacuation of hematoma planned within 24 h after the onset and secondary intracerebral hemorrhage. More detailed description of the study inclusion and exclusion criteria can be gotten in Table 1.

**Table 1:** Study inclusion and exclusion criteria

*Inclusion criteria*
Ages eligible for study: 18 years or older
Genders eligible for study: both
AICH confirmed by craniocerebral CT scan
Within 6 h after the onset of symptom
GCS ≥ 6
Sign the informed consent form
*Exclusion criteria*
Secondary intracerebral hemorrhage resulting from trauma, brain tumor, blood diseases, arteriovenous malformation or aneurysm, etc.
Patients with severe heart, liver or kidney disease
Intolerance to herbal medicine
Patients with allergies
Patients planning a surgical evacuation of hematoma with severe cerebral hernia at super-early stage
Patients with poor compliance

At screening, this criteria start. *CT* computed tomography, *GCS* Glasgow Coma Scale, *TCM* Traditional Chinese Medicine, *AICH* acute intracerebral hemorrhage

### Randomization

We define the herbal medicine as PBC or RBS under the criteria of Chinese Pharmacopoeia of 2010 version. The combined herbal drugs, such as relieving heat and calming liver Yang, decreasing wind and dispersing phlegm, loosing the bowels, are also under the criteria of Chinese Pharmacopoeia of 2010 version. Within 6 h from the onset, participants will be assigned at random to one of three treatment groups with a ratio of 1:1:1: (1) Group A: RBS, stasis-breaking herbal medicine (8 herbals); (2) Group B: PBC, herbal medicine without stasis-breaking herbal medicine (6 herbals) and (3) Group C: placebo comparator, only placebo. Randomization is generated and stratified in blocks of six by PROC PLAN process using the SAS software version 9.13. Stratified block randomization is concealed using a sequentially numbered and opaque envelope. The number of each group will be in balance within research centers and by the baseline NIHSS severity (≤17 vs. >17), age (>18 years) and hemorrhagic location (basal ganglia vs. all other), patients’ sex, baseline Glasgow Coma Scale, duration from onset to the first CT scan.

### Intervention

#### Treatment

The interventions in each group are listed in the Table 2. Treatment is administered within 6 h after the onset. The experimental drugs are given as powder prepared by Kangyuan Pharamaceutical factory according to a Good Manufacture Practice (GMP). Each unit of TCM medicine or placebo is dissolved into solution in a cup of boiling water of 200 ml. Each patient orally takes 200 ml two times daily for 10 days. And the placebo medicine is provided according to the same standardized method. Quality control is enforced strictly throughout the trial.

**Table 2:** Groups and interventions

Groups	Interventions	Signa
RBS	8 herbals [with 2 herbals of RBS (*Hirudo* and *Tabanus*), as well as herbals of PBC and other combined herbals]	One dose, bid, by oral or nasogastric tube for 10 days
PBC	6 herbals (remove 2 herbals of RBS, left with PBC and other combined herbals)	One dose, bid, by oral or nasogastric tube for 10 days
Placebo comparator	Placebo herbal medicine (with dextrin, farina and so on)	One dose, bid, by oral or nasogastric tube for 10 days

*RBS* removing blood stasis, *PBC* promoting blood circulation

### CT scanning

At screening, the baseline CT scans are obtained immediately after arriving at treating hospital to identify intracerebral hemorrhage; the first follow-up CT scans are done at 24 h after the onset of symptoms (range 21–27) to assess hematoma growth; and the second follow-up CT scans are performed on 10 days (range 8–14) after drug administration. The hematoma volume is measured by ABC/2 Coniglobus formula (Kothari et al. 1996). Hematoma growth is operationally defined as an increase in the hematoma volume of >33 % as measured by image analysis on the 24 h CT in comparison with the baseline CT scan (Kothari et al. 1996) (if the first follow-up CT scan cannot be done within 24 h from the onset, a 48-h CT scan will be analyzed). Blinding for the two neuroradiologists is maintained through analyzation of digital CT data. Computerized planimetric techniques are used to calculate the hematoma volume to valuated the primary end point. In a previous study, the interobserver agreement of this method has been proven perfect (Xu et al. 2015).

### Follow-up and outcome evaluation

Clinical assessments are performed at enrollment; at 24 h after the onset of symptoms; on the 10th day after admission; and on the 90th day after the stroke. During the follow-up period, the following scales will used:the GCS assessing the level of consciousness according to the score ranging from 15 (normal) to 3 (deep coma);the NIHSS measuring neurologic deficit according to the score ranging from 0 (normal) to 42 (coma with quadriplegia), the mRS evaluating functional independence, the BI assessing the ability of daily life. More detailed information about the schedule of study visits is shown in Table 3.

**Table 3:** Study visits

Assessment method	Screening	Onset (0 h)	24 h	10–14 days	90-day follow-up
Patient population	W	–	–	–	–
History	W	–	–	–	–
Physical examination	W	W	–	–	–
CT/CTA	W		W	W	–
Confirmation of entry criteria	W	–	–	–	–
Signing consent form	W	–	–	–	–
Serum and urine test	W	–	–	–	–
Concomitant medication or treatment	W	W	W	W	W
GCS	W	W	W	W	–
NIHSS	W	W	W	W	W
BI	W		–	W	W
mRS	–	–	–	W	W
Adverse events	w	w	w	w	W
Summary of patient	–	–	–	–	W

*CT* computed tomography, *CTA* computed tomography angiography, *NIHSS* National Institutes of Health Stroke Scale, *mRS* modified Rankin Scale, *BI* Barthel Index, *GCS* Glasgow Coma Scale

### Adverse event validation

All adverse events will be recorded during the stay in hospital and all serious adverse events will be recorded 90 days after onset. Clinical events committee, which are composed of four expert physicians independent of the research centers, will validate all adverse events. Relatedness categories are as follows: (1) primary disease-related: events obviously due to primary disease with no time relationship to the therapy, or medication. (2) concomitant disease-related: events due to diseases instead of the primary disease with no time relationship to therapy or medication; (3) TCM medicine-related: events obviously due to TCM medicine with no time relationship to other therapy;(4) medicine unknown: medicine related but unable to attribute a specific medicine; (5) individual difference-related: events have strong time relationship to individual constitution, whose allergic constitution are intolerant to traditional Chinese medicine (TCM); (6) other; and (7) unknown.

### Primary outcome

The primary end-point measure is the percent change in the hematoma volume measured by ABC/2 Coniglobus formula (Kothari et al. 1996) at the 24th hour after the onset. Hematoma growth is operationally defined as an increase in the hematoma volume of >33 % as measured by image analysis on the 24 h CT compared with the baseline CT scan.

### Secondary outcomes

Three secondary clinical efficacy outcomes are included as follows: (1) death owing to any cause on the 14th day; (2) disability as defined by mRS score ≤2 on the 90th day; and (3) change in NIHSS score at the 24th hour after the onset of symptoms.

In the study two technical efficacy outcomes are included as follows: (1) volume of hematoma growth as measured by a CT scan at 24 ± 3 h after onset of symptoms compared with the baseline CT scan; (2) recurrent bleeding measured by CT scan on 10 days after drug administration.

Two study safety outcomes are included as follows: (1) all serious adverse events; (2)hematoma growth at 24 ± 3 h.

### Blinding

Before the code is broken following receipt of e-mail notification of the completion of the study, the persons involved in the research including the investigators, subjects and the data analysts are blinded to the interventions and outcomes. Only the data administrators are permitted access to unblinded data.

## Statistical considerations

### Sample size

The primary efficacy outcome is the percent change in the volume of hematoma at the 24th hour after onset in the two treatment groups as compared with the placebo group, assessed via the blinded measurement of hematoma by ABC/2 Coniglobus formula. The statistical hypothesis on the percentage of hematoma expansion is that the relative hematoma volume in subjects of three groups are the same to each other. Power and sample size are determined using relative hematoma volume at 24 h as the main outcome variable. According to the previous literature (Huang et al. 2010; Wang et al. 2013; He et al. 2002), the true proportions of subjects in 3 groups with different rations of hematoma expansion at 24 h are presented in Table 4. In this case, the $\upgamma$ is 12.65, $\uppi\_\hbox{max}$ 38 % and $\uppi\_\hbox{min}$ 7 %. With a two-sided alpha level at 0.05, 285 participants finishing the final follow up provide 90 % power to test the hypothesis of the study’s primary effectiveness, which is calculated by the following formula; assuming that the non-investigation rate of the primary outcomes is 20 %, 360 participants will be needed.

**Table 4:** Hypothesized true outcomes for sample size calculations

Randomized group	PBC	RBS	Placebo
Percentage of change	7	16	38

*RBS* removing blood stasis, *PBC* promoting blood circulation

$$n = 1641.6\upgamma/((\sin^{ \wedge } ( - 1)\surd (\uppi\_\hbox{max} )) - \sin^{ \wedge } ( - 1)\surd\uppi\_{\rm min}^{ \wedge } 2$$

### Statistical analyses

All analysis are by intention to treat with a beta level of 0.10 and an alpha level of 0.025 (Type I and Type II error). The primary outcome is the percent change in the volume of the hematoma measured by a CT scan at 24 ± 3 h after the onset compared with the baseline CT scan. The volumes of the hematoma measured by a CT scan will be analyzed using generalized linear mixed models. The subjects and the readers (two neuroradiologists) will be defined as random effects, and the baseline volume of hematoma, the time from the onset to CT scanning, and the time from CT scanning to study intervention will be defined as fixed-effect covariates. The percentages of changes in the volume of hematoma are transformed to normality and eliminate negative values via logarithmic transformations. Comparisons will be made between the two treatment groups with placebo using Bonferroni’s method with a threshold of significance of 0.0167. The threshold of significance for all other comparisons was 0.05. Subjects died before finishing the follow up will be assigned the worst possible scores for measures of neurologic deficit and function prognosis. Regarding surviving subjects with missing outcome data, the last visit will be carried down. The scores on mRS will be analyzed in a cumulative logit model, with statistical adjustment for age, the baseline hematoma volume, the site of hemorrhage, and the baseline neurological status. The scores on the BI and NIHSS will be analyzed with Wilcoxon rank-sum tests. The difference of the frequency of SAE in the three groups at 3 months will be analyzed with Fisher’s exact test or the Chi square test. All dates will be analyzed via SPSS software, version 21.0.

### Lost-to-follow-up and missing data

It is supposed that there is no missing safety data on SAE, because of the careful observation and detailed records while in hospital. For surviving patients with missing outcome data, the last observation was carried forward. Regarding other incomplete primary outcome data, we will use multiple imputation to fill in missing data with standard statistical procedure.

### Data and safety monitoring

The Data Safty Monitoring Committee deliberates the data from intermediate safety analysis report and contacts the Trial Steering Committee. Interim results are extremely secret and once one of the three groups shows a distinct advantage over others, the committee will counsel to end the study ahead of schedule.

## Rationale for the design

### Justification for the choice of three groups

In the study, we design three groups to verify the better efficacy of combining RBS with PBC herbal medicine based on formula components analysis/methods. As recorded in the Chinese Pharmacopoeia of 2010 version (Chinese Pharmacopoeia Commission 2010), RBS herbal medicine should be combined with PBC and other herbals correctly in the Traditional Chinese Medicine Formula in order to avoid the rebleeding risk. Some herbal have strong RBS and promoting blood circulation function, for example, *leech* can cause rebleeding (Liu et al. 2012). Some others have two-way adjustment pharmacological effect, for example, *Radix notoginseng* can not only promote blood circulation but also stop bleeding. Traditionally, the prescription of Chinese medicine to learn is through reasonable compatibility other than a single drug, to eliminate this rebleeding risk.

### Justification for the choice of the therapeutic time window

The decision on the time of beginning PBS therapy within 6 h after the onset of symptoms is made on the basis of the data from previous studies (Xu et al. 2015). Among most (83 %) of patients with hematoma growth done the first-time CT scan within 6 h after the onset, enlargement after 24 h of onset happens rarely (Kazui et al. 1996). So some neurologists show their opinion that PBC and RBS herbal medicine should be used after 24 h of onset in order to prevent rebleeding risk (Wanzhng et al. 2004). However others support that PBC and RBS herbal medicine should be administrated as sooner as possible (Li 2003). And furthermore, Guo et al.’s (2005) previous study did not show deterioration of condition of the AICH patients who were treated with herbal compound within 6 h time window from the onset, suggesting better curative effect for earlier therapy. This finding might reduce hospital stay, healthcare-related expenses, as well as complications and mortality.

## Discussion

Further prospective, multicenter, randomized, placebo control clinical trials are needed to provide better quality evidence, though in a retrospective study (Xu et al. 2015), we found that PBC and RBS herbal medicine were not associated with an increased risk of hematoma growth within 24 h after the onset of symptoms. Meanwhile, a meta-analysis (Li et al. 2015) which included 9 randomized-controlled clinical trials with 798 individuals showed that PBC and RBS single therapy and combination therapy for acute ICH could reduce the volumes of brain hematoma and cerebral edema, improve the neural function and reduce the mortality and disability rate. Moreover, unlike western conventional medication group, fewer adverse reactions occurred in herb medicine group. In spite of the obviously positive results, It’s too early to draw conclusion about the efficacy and safety of PBC & RBS for ICH since the limitation of the included studies in the meta-analysis. Therefore, high-quality RCTs are needed.

The CRRICH Trial will provide definitive information on the efficacy and safety of the RBS therapy. The trial is designed to learn lessons from study of earlier conservative therapies that cannot demonstrate the treatment benefit of TCM with RBS rather well. Unlike those prior studies (Fan and Jin 2000), the trial set three groups (PBS & RPS & Placebo), better observating the efficacy of RBS or PBS to confirm whether PBC and RBS herbal medicine induce the incidence of hematoma enlargement of AICH patients within the 6 h time window from onset. The shortages of prior studies are as follows: (1) most studies are retrospective, unblinded trials; (2) mixed PBS with RBS; (3) time window from onset is 24 h or later; (4) not multicenter RCT. In order to make up for these limitations, we have designed a prospective, 13 hospitals, randomized, placebo-controlled clinical trial (clinicaltrials.gov: NCT01918722) to confirm if PBC and RBS herbal medicine induce the incidence of hematoma enlargement of AICH patient within the 6 h time window from onset. The trial enrolled its first patient on October 25, 2013. And 280 cases have recruited so far. The study is still ongoing until all 360 patients are completed in December 2016. When completed, we will provide pivotal data allowing.

## Abbreviations

ICH: intracerebral hemorrhage
CT: computed tomography
CTA: computed tomography angiography
NIHSS: National Institutes of Health Stroke Scale
mRS: modified Rankin Scale
BI: Barthel Index
GCS: Glasgow Coma Scale
SATCM: State Administration of Traditional Chinese Medicine of the P.R.C.
TCM: traditional Chinese medicine
RBS: removing blood stasis
PBC: promoting blood circulation
